# Hantavirus L protein exhibits shutoff activity mediated by its N-terminal endonuclease domain

**DOI:** 10.1038/s41598-026-47692-3

**Published:** 2026-04-14

**Authors:** Kohei Oishi, Tomoki Yoshikawa, Satoshi Taniguchi, Madoka Kawahara, Takeshi Kurosu, Masayuki Shimojima

**Affiliations:** Department of Virology I, National Institute of Infectious Diseases, Japan Institute for Health Security, Tokyo, 208-0011 Japan

**Keywords:** Shutoff, Endonuclease, Cap-snatching, Hantavirus, L protein, Biochemistry, Microbiology, Molecular biology

## Abstract

The hantavirus L protein is a viral polymerase essential for viral transcription and replication; however, its expression in mammalian cells has been notoriously difficult. In this study, we achieved robust plasmid-based expression of the L protein by combining a T7-driven system with mutations that reduce endonuclease activity. This strategy was pivotal, as conventional RNA polymerase II (Pol II)-dependent systems failed to yield detectable expression. Although wild-type L protein was barely detectable, its presence suppressed co-expressed genes, suggesting a potent host shutoff activity that inhibits both trans-gene and its own (cis-) expression. Leveraging functional homology to the influenza PA-X protein, we identify amino acid residues essential for the shutoff activity of L protein by using its N-terminal fragment, which can be expressed via standard Pol II-dependent systems. Our mutagenesis analysis established a toolset for the predictable fine-tuning of shutoff activity and L protein expression levels, facilitating a detailed analysis of the interplay between polymerase activity and viral replication. These findings elucidate the mechanisms underlying the difficulty in expressing the hantavirus L protein and emphasize the necessity of accounting for these *cis-*, and *trans-*regulatory effects in functional analyses, such as in minigenome assays, to prevent data misinterpretation.

**Supplementary Information:**

The online version contains supplementary material available at 10.1038/s41598-026-47692-3.

## Introduction

Hantaviruses are rodent-borne zoonotic viruses transmitted to humans, causing hantavirus pulmonary syndrome (HPS) and hemorrhagic fever with renal syndrome (HFRS), with mortality rates of up to 38–50% and 15%, respectively^1,2^. As members of the *order Bunyavirales*, hantaviruses possess a tri-segmented, negative-sense, single-stranded RNA genome consisting of L, M and S segments^3,4^. The L segment encodes a 250-kDa L protein, which contains an RNA-dependent RNA polymerase (RdRp) domain in the center and possesses various enzymatic functions involved in transcription and replication^5^. The M segment encodes a glycoprotein precursor that is co-translationally cleaved into the envelope proteins Gn and Gc^6^. The S segment encodes the nucleoprotein (N), which encapsidates the viral genome^4^. Additionally, the S segments of several hantaviruses encode a nonstructural protein (NSs) that potentially modulates the host immune response^7^.

The hantavirus mRNAs contain heterologous nucleotides at their 5’ ends, indicating that the virus steals the cap structure from cellular mRNAs to initiate its own mRNA synthesis, a process called cap-snatching^8^. Cap-snatching involves the recognition of capped cellular mRNAs by a viral cap-binding domain, and the cap is cleaved off several nucleotides downstream by a viral endonuclease^9,10^. In influenza A virus, the most extensively characterized model for cap-snatching, these functions are distributed across distinct subunits: PB2 subunit contains the cap-binding domain, PA subunit harbors the endonuclease domain, and PB1 subunit possesses RdRp activity^11^. In contrast, hantaviruses employ a single L protein that is thought to encompass all three functions within a single, large polypeptide^5,12^.

Despite their structural complexity and high molecular weight, the structures of hantavirus L proteins have been determined in recent years^5,12,13^. An endonuclease motif belonging to the PD-D/ExK superfamily of cation dependent nuclease has been identified at the N-terminus of hantavirus L proteins^5,12,13^. Reflecting its potent catalytic activity, the expression and purification of L proteins in heterologous systems, such as insect cells and *Escherichia coli*, frequently require the inactivation of the endonuclease domain to ensure sufficient yield for structural studies^5,13–15^. Consistent with these observations, several reports have highlighted that the wild-type (WT) L protein remains difficult to detect in mammalian cells, even when its expression is driven by strong promoters in plasmid-based systems^16^. This difficulty may result from either the inherent cellular toxicity or the intrinsic instability of the L protein itself; however, the precise mechanism remains to be clarified.

In this study, we demonstrated that the expression of hantavirus L protein from plasmids in mammalian cells is specifically achieved through a T7-driven system rather than by standard RNA polymerase II (Pol II)-dependent promoters such as CMV. While the expression of WT L was barely detectable, its presence induced the potent suppression of co-expressed reporter genes, suggesting that the hantavirus L protein possesses a shutoff activity that inhibits gene expression, including its own. Consistent with this, our mutagenesis analysis demonstrated that the N-terminal endonuclease activity of L protein correlates inversely with its own expression levels. These finding elucidate the inherent difficulty in expressing and detecting the hantavirus L protein.

## Results

**Fig. 1:**
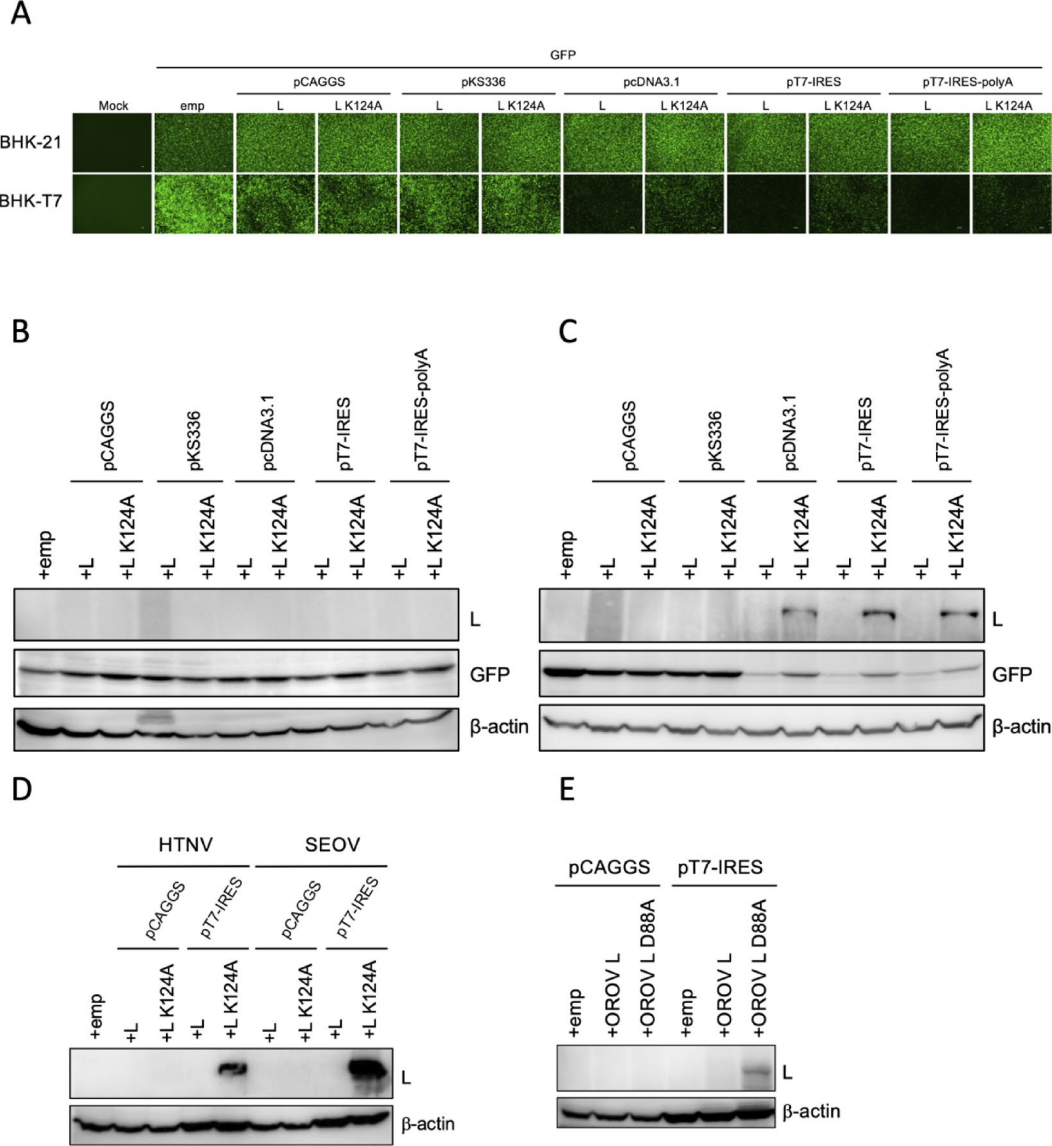
Plasmid-based hantavirus L protein expression is achieved by combining a T7-driven system with mutations that reduce endonuclease activity. (**A**) Fluorescence microscopy of GFP co-expressed with wild-type (WT) HTNV L or the L K124A mutant. BHK-21 or BHK-T7 cells were co-transfected with pCAGGS-GFP or pT7-IRES-GFP plasmid, together with the indicated expression plasmid encoding either WT HTNV L or the L K124A mutant. GFP signals were observed at 48 h post-transfection (hpt). (**B** and **C**) Western blot analysis of HTNV L and GFP expression. BHK-21 (**B**) and BHK-T7 (**C**) were co-transfected with GFP-expressing plasmids along with the indicated plasmids encoding the WT or the K124A mutant of HTNV L. At 48 hpt, cell lysates were subjected to immunoblotting. HTNV L proteins, fused with a C-terminal FLAG tag, was detected using an anti-FLAG antibody. (**D**) Expression of HTNV L and SEOV L protein. BHK-T7 cells were transfected with either pCAGGS or pT7-IRES plasmid encoding the WT or the K124A mutant L proteins of HTNV or SEOV. (**E**) Expression of OROV L protein. BHK-T7 cells were transfected with pCAGGS or pT7-IRES plasmids encoding WT or mutant (D77A) OROV L. Cell lysates were analyzed by western blotting at 48 hpt. b-actin served as a loading control.

*Plasmid-based hantavirus L protein expression is achieved by combining a T7-driven system with mutations that reduce endonuclease activity*. Despite its essential role, hantavirus L protein expression in mammalian cells has been notoriously difficult^16^. To determine the optimal conditions for expressing it, we constructed various plasmid backbones encoding the Hantaan virus (HTNV) L protein with distinct properties, including different promoters and the presence or absence of splicing signals and poly(A) tails (Table 1). To examine whether the HTNV L endonuclease inhibits its own expression, we also generated plasmids encoding an endonuclease-deficient mutant L protein (L K124A) by introducing a mutation into its catalytic site^13^. Cells were co-transfected with a plasmid encoding either the HTNV WT or mutant L protein, along with a GFP-expressing plasmid as a transfection control (Fig. 1A). When these plasmids were transfected into BHK-21 cells, neither WT nor the mutant L protein was detectable by western blotting (Fig. 1B). However, robust GFP expression from the co-transfected plasmid was confirmed via fluorescence microscopy and western blotting (Fig. 1A and B), verifying that transfection efficiency was sufficient. Next, to evaluate a T7-driven system for HTNV L protein expression, BHK-T7 cells stably expressing T7 RNA polymerase were utilized. In contrast to the results in BHK-21 cells, transfection of T7 promoter-driven plasmids into BHK-T7 cells yielded detectable levels of the L K124A mutant, whereas the WT L protein remained undetected (Fig. 1C). These results indicate that a T7-driven system, rather than standard Pol II-dependent expression systems, is suitable for the expression of the full-length L protein. Since L K124A was expressed in BHK-T7 cells but not in BHK-21 cells, using pcDNA3.1, pT7-IRES, and pT7-IRES-polyA plasmids, which share a T7 promoter but differ in other regulatory elements, it appears that splicing signals and poly(A) tails do not significantly affect HTNV L protein expression (Fig. 1B, C and Table 1). Although WT L expression remained below the detection limit even in the T7-driven system, its presence suppressed the expression of co-transfected GFP (Fig. 1B and C). These data suggest that the HTNV L protein was indeed expressed by the T7-driven system but suppressed the expression of both GFP and the L protein itself through its shutoff activity.

**Table 1:** Characteristic of HTNV L expression plasmids.

Vector	Promoter	Splicing signals	Poly(A) tails
pCAGGS	CMV	+	+
pKS336	EF-1a	+	+
pcDNA3.1	CMV/T7	–	+
pT7-IRES	T7	–	–
pT7-IRES-polyA	T7	–	+

To examine whether plasmid-based expression of L proteins from other members of the *class Bunyaviricetes* also requires a T7-driven system, we evaluated the expression of L proteins from Seoul virus (SEOV), an Old World hantavirus^17^, and Oropouche virus (OROV), an orthobunyavirus^18^. The expression of their endonuclease-deficient mutants, SEOV L K124A and OROV L D88A, was achieved using the T7-driven system but not via Pol II-dependent promoters (Fig. 1D and E). Under both conditions, WT L protein expression remained undetectable for either virus (Fig. 1D and E). These findings are consistent with our observations for HTNV L, suggesting that a T7-driven system is suitable for the expression of certain bunyavirus L proteins and that their N-terminal endonuclease activity suppresses their own expression.

**Fig. 2:**
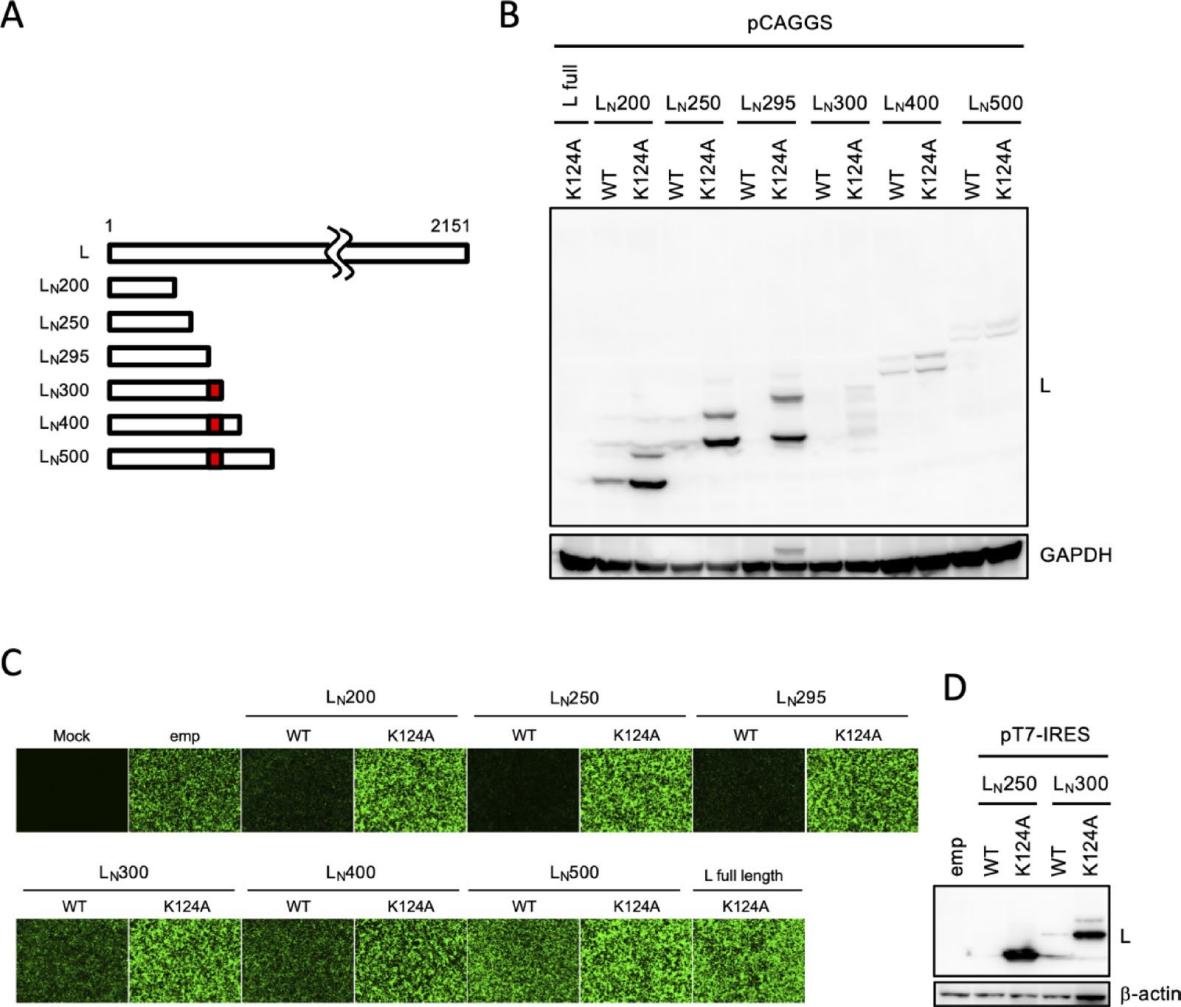
Identification of the region restricting hantavirus L protein expression from CMV promoter-driven plasmids. (**A**) Schematic representation of HTNV L N-terminal fragments. C-terminal deletion mutants, designated as L_N_200, L_N_250, L_N_295, L_N_300, L_N_400, and L_N_500, comprise the N-terminal 200, 250, 295, 300, 400, and 500 amino acids of the L protein, respectively. The red bar indicates a specific region associated with the low expression efficiency when using the pCAGGS vector. (**B**) Expression of full-length L and its N-terminal fragments. 293 T cells were transfected with pCAGGS plasmids encoding the full-length or indicated N-terminal fragments of HTNV L protein. Cell lysates were subjected to immunoblotting at 48 hpt. GAPDH served as a loading control. (**C**) Fluorescence microscopy of GFP co-expressed with HTNV L N-terminal fragments. 293 T cells were co-transfected with pCAGGS-GFP, together with a pCAGGS plasmid encoding indicated WT or K124A mutant of N-terminal fragments of HTNV L protein. GFP signal was visualized at 48 hpt. (**D**) Expression of L_N_250 and L_N_300 from pT7-IRES plasmids. The expression levels of WT or K124A mutant of L_N_250 and L_N_300 were detected at 48 hpt in BHK-T7 cells.

*Identification of the region restricting hantavirus L protein expression from CMV promoter-driven plasmids*. To identify the specific region within the L protein that restricts its expression in Pol II-dependent expression systems, we constructed a series of C-terminal deletion mutants for both WT and K124A HTNV L backgrounds (Fig. 2A). The K124A mutant was introduced to circumvent endonuclease-mediated self-downregulation. When 293 T cells were transfected with pCAGGS plasmids encoding C-terminal deletion mutants of the L protein, protein expression remained generally limited, although faint bands were observed for L_N_200, L_N_400 and L_N_500 (Fig. 2B). Notably, while the full-length L K124A remained undetectable from the pCAGGS vector, expression was achieved for shorter fragments consisting of the N-terminal 200, 250, and 295 aa (Fig. 2B). In contrast, fragments exceeding 300 aa in length were not detectable (Fig. 2B). Additional bands were observed above the expected bands during western blot analysis of the C-terminal tags for reasons that remain unclear (Fig. 2B). Furthermore, when the C-terminal deletions of WT L protein were co-expressed with GFP, short N-terminal fragments such as L_N_200, L_N_250 and L_N_295 markedly suppressed the GFP signal (Fig. 2C). Because the introduction of the K124A mutation into these fragments abolished this repression and enhanced their own expression levels (Fig. 2B and C), these results suggest that the N-terminal region of the L protein possesses shutoff activity that suppresses the expression of both other genes and itself. In contrast, WT L fragments of 300, 400, and 500 aa exhibited only weak inhibitory effects on the co-expressed GFP signal (Fig. 2C). This was likely attributable to inherently low production levels rather than self-suppression via their shutoff activities, as even the shutoff-deficient L K124A 300 aa fragment remained poorly expressed (Fig. 2B). To investigate whether the inherently low expression of the 300 aa L fragment results from protein instability or other intrinsic factors, we attempted to express it using the T7-driven system. In contrast to the results with pCAGGS, the pT7-IRES plasmid successfully expressed the shutoff-deficient fragments, L_N_250 K124A and L_N_300 K124A. On the other hand, the expression of L_N_250 and L_N_300 was undetectable due to self-suppression mediated by their shutoff activities (Fig. 2D). These results indicate that the region between residues 295 and 300 is critical for the poor expression of the HTNV L protein from the pCAGGS vector.

**Fig. 3:**
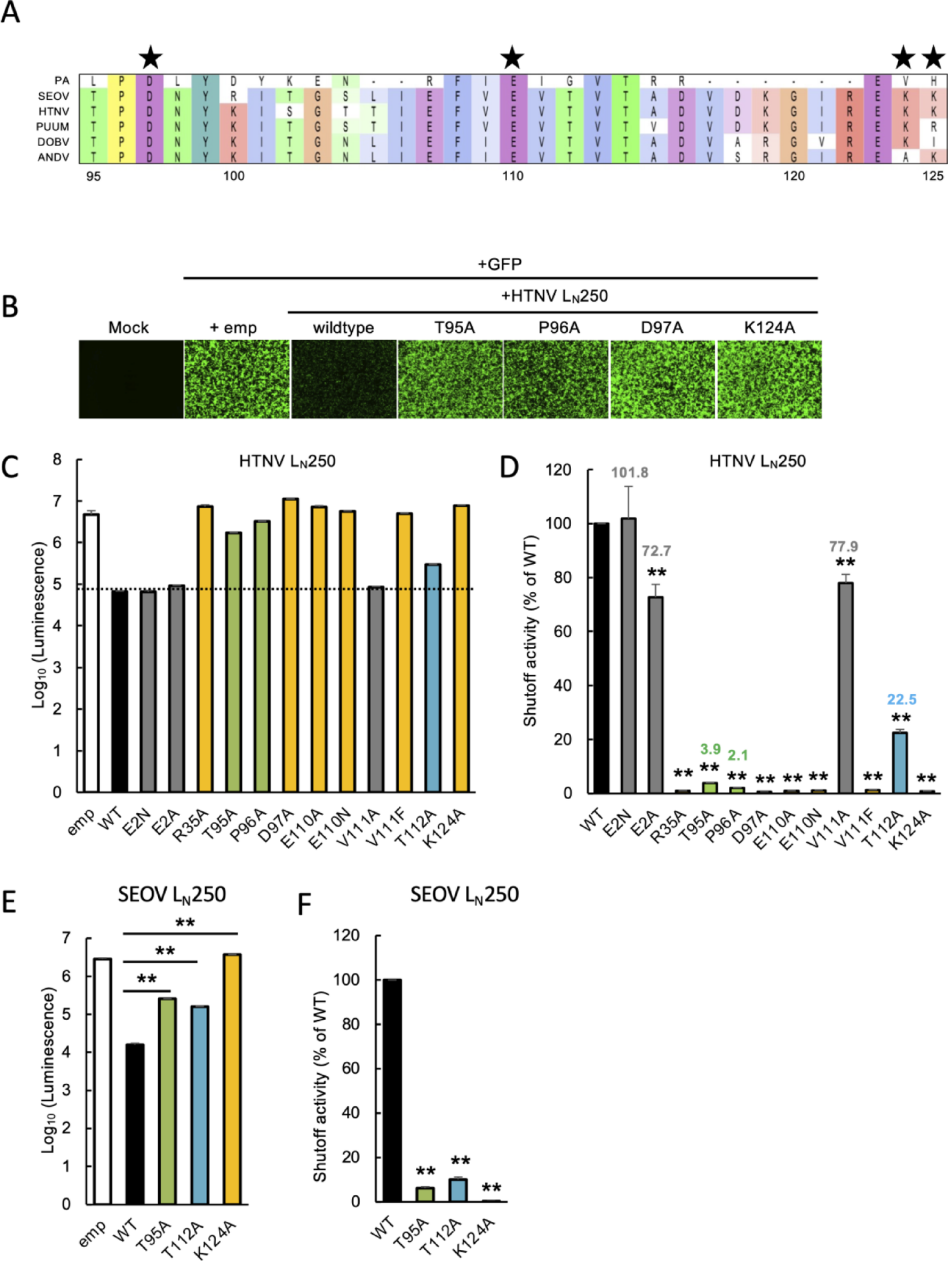
Identification of the amino acid residues required for the shutoff activity of the hantavirus L protein N-terminal domain. (**A**) Comparison of the amino acid sequences of N-terminal endonuclease domain from various viruses. Amino acid sequences of influenza A virus PA, L proteins of Old World hantaviruses including HTNV, SEOV, PUUV, and DOBV, and the new world hantavirus, Andes virus (ANDV), were aligned. Stars indicate amino acid residues in hantavirus L proteins that correspond to the endonuclease motif PD-D/ExK. (**B**) GFP signals from 293 T cells co-expressing WT or the indicated mutants of HTNV L_N_250. (**C**) Luminescence of firefly luciferase co-expressed with WT or mutants HTNV L_N_250. 293 T cells were co-transfected with a firefly luciferase-expressing plasmid along with either an empty vector, or a plasmid encoding either WT or mutant L_N_250. Luminescence was measured at 48 hpt using the ONE-Glo Luciferase Assay System and a luminometer. Luminescence data are presented as means ± SD (*n* = 3 technical replicates). (**D**) Relative shutoff activities of mutants HTNV L_N_250. Shutoff activity of WT HTNV L_N_250 was set to 100%. The values above the bars represent the relative shutoff activity of each mutant. The shutoff activity shown is the mean values ± SD (*n* = 3 technical replicates). **, *P* < 0.01 (one-way ANOVA followed by Dunnett’s test). (**E**) Luminescence of luciferase co-expressed with WT or mutants SEOV L_N_250. Luminescence data are presented as means ± SD (*n* = 3 technical replicates). **, *P* < 0.01 (one-way ANOVA followed by Tukey’s test). (**F**) Shutoff activities of WT or mutants SEOV L_N_250. Shutoff activity of WT SEOV L_N_250 was set to 100%. The shutoff activity shown is the mean values ± SD (*n* = 3 technical replicates). **, *P* < 0.01 (one-way ANOVA followed by Dunnett’s test).

*Identification of the amino acid residues required for the shutoff activity of the hantavirus L protein N-terminal domain*. We demonstrated that N-terminal fragments of the HTNV L protein shorter than 300 aa could be expressed from the pCAGGS plasmid and exhibited shutoff activity (Fig. 2B and C). Based on this finding, the N-terminal fragment L_N_250, was utilized in a screening assay to identify the amino acid residues required for the shutoff activity of the L protein. Since the shutoff activity of the L protein depends on its endonuclease activity, we aligned the sequences around the conserved PD-D/ExK motif. We confirmed that this region is highly conserved across both Old World hantaviruses including HTNV, Seoul virus (SEOV), Puumala virus (PUUV), and Dobrava-Belgrade virus (DOBV), and New World hantaviruses, such as the Andes virus (ANDV) (Fig. 3A). Notably, the PA subunit of the influenza A virus, which utilizes an endonuclease for the cap-snatching in a manner similar to that of the hantavirus L protein^11^, also possesses these conserved key residues (Fig. 3A). To examine whether the endonuclease active site is essential for the shutoff activity of L_N_250, amino acid residues within and surrounding the PD-D/ExK motif were substituted with alanine. While the co-expression of L_N_250 strongly suppressed GFP expression, mutations such as T95A, P96A, D97A and K124A completely abolished the shutoff activity (Fig. 3B), further supporting that the shutoff activity of L_N_250 relies on its endonuclease activity.

Influenza A virus expresses the PA-X protein from the mRNA encoding PA via a ribosomal frameshift^19^. Although PA and PA-X share an identical N-terminal endonuclease domain which is essential for the shutoff activity, PA-X exhibits significantly stronger shutoff activity than PA^20^. Previous studies have extensively mapped the amino acid residues required for the shutoff activity of PA-X, revealing that essential residues are located not only within and surrounding the endonuclease domain but also in regions relatively distant from the active site^21^. Based on these findings and the structural conservation between the influenza PA (PA-X) and the HTNV L protein^10^, we utilized L_N_250 to introduce mutations at residues corresponding to those essential for PA-X activity and evaluated their impact on the shutoff function of L protein. To assess the shutoff activity of L_N_250 or its mutants, a plasmid encoding firefly luciferase was co-transfected with either an empty vector or an L_N_250-expressing plasmid. Notably, many of the amino acid residues corresponding to those important for PA-X activity, such as R35 and E110, as well as the catalytic residue D97, were also essential for the shutoff activity of L_N_250 (Fig. 3C, yellow). Interestingly, while the substitution of E110 with either alanine (A) or asparagine (N) abolished the shutoff activity, the V111A mutation significantly reduced it, whereas the V111F mutation had little impact on shutoff activity of L_N_250 (Fig. 3C). Alanine substitutions at T95, P96, and T112 of L_N_250 significantly, though not completely, reduced its shutoff activity (Fig. 3C, green and blue). Moreover, while the second amino acid of PA-X is required for its shutoff activity via N-terminal acetylation^22^, the corresponding substitutions in L_N_250 (E2N and E2A) had little impact on its shutoff activity (Fig. 3C, gray). For quantitative comparison of the shutoff activity of each mutant L_N_250, relative shutoff activity was determined by dividing the luminescence value of firefly luciferase co-transfected with an empty vector by that co-transfected with L_N_250 expressing plasmids (Fig. 3D). The majority of the L_N_250 mutants exhibited near-complete loss of shutoff activities, measuring less than 1.5% of that of WT L_N_250 (Fig. 3D, yellow). The T95A and P96A mutants also showed significantly reduced shutoff activities, retaining 3.9% and 2.1% of the WT level, respectively (Fig. 3D, green). In contrast, the T112A showed a partial reduction, maintaining 22.5% of the WT L_N_250 shutoff activity (Fig. 3D, blue). Furthermore, although E2A and V111A appeared to have shutoff activities comparable to WT (Fig. 3C), their calculated shutoff activities were, 72.7% and 77.9%, respectively (Fig. 3D, gray). From these results, we identified amino acid residues of HTNV L_N_250 involved in its shutoff activity.

To determine whether the identified amino acid residues similarly affect the shutoff activity of other hantaviruses, we selected three mutations representing distinct levels of shutoff activity in HTNV L_N_250: T95A, T112A, and K124A. These mutations were introduced into the corresponding positions of the SEOV L_N_250 to evaluate their functional conservation. Consistent with the results for HTNV L_N_250, the K124A mutation led to a complete loss of shutoff activity of SEOV L_N_250, while T95A and T112A retained modest and relatively high shutoff activity, respectively (Fig. 3E and F). These data indicate that the identified amino acid residues mediating the shutoff activity of L_N_250 are functionally conserved across hantavirus species. Furthermore, the specific mutations identified here provide a robust toolset for modulating the shutoff activity of L_N_250 at distinct, predictable levels.

**Fig. 4:**
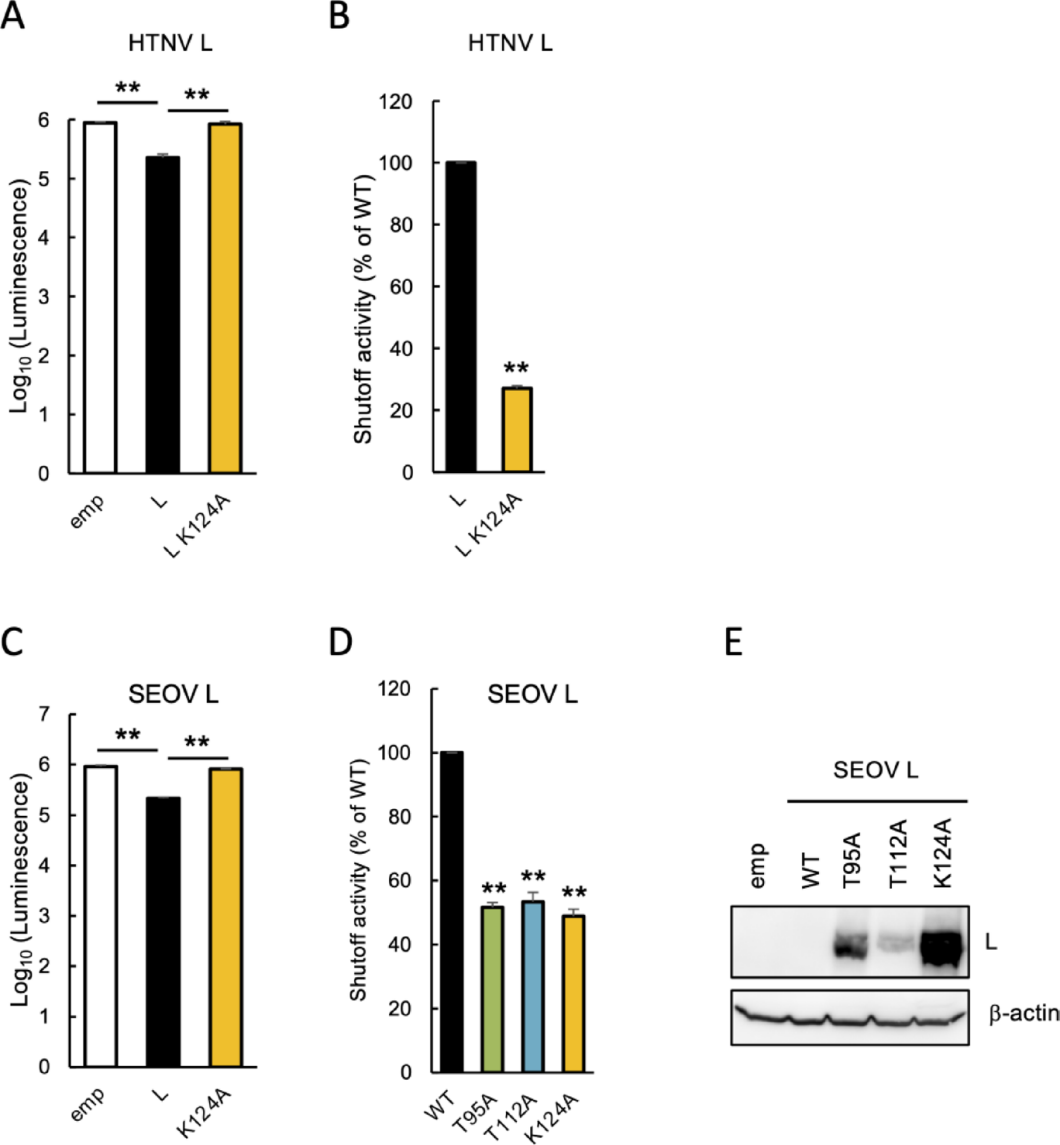
Hantavirus L protein exhibits shutoff activity dependent on its N-terminal endonuclease domain. (**A**) Luminescence of firefly luciferase co-expressed with WT or K124A mutant HTNV L. BHK-T7 cells were co-transfected with a firefly luciferase-expressing plasmid, together with either an empty vector, or a pT7-IRES plasmid encoding either WT or K124A mutant HTNV L protein. Luminescence was measured at 48 hpt using the ONE-Glo Luciferase Assay System. Luminescence data are presented as means ± SD (*n* = 3 technical replicates). *P* < 0.01 (one-way ANOVA followed by Tukey’s test). (**B**) Shutoff activities of WT or K124A mutant HTNV L. Shutoff activity of WT HTNV L was set to 100%. The shutoff activity shown is the mean values ± SD (*n* = 3 technical replicates). **, *P* < 0.01 (two-tailed unpaired Student’s *t*-test). (**C**) Luminescence of luciferase co-expressed with WT or K124A mutant SEOV L in BHK-T7 cells. Luminescence data are presented as means ± SD (*n* = 3 technical replicates). **, *P* < 0.01 (one-way ANOVA followed by Tukey’s test). (**D**) Shutoff activities of WT or indicated mutants of SEOV L. Shutoff activity of WT SEOV L was set to 100%. The shutoff activity shown is the mean values ± SD (*n* = 3 technical replicates). **, *P* < 0.01 (one-way ANOVA followed by Dunnett’s test). (**E**) Expression of WT or mutant SEOV L protein. BHK-T7 cells were transfected with a pT7-IRES plasmid encoding either WT or mutant SEOV L protein. Cell lysates were analyzed by western blotting at 48 hpt. b-actin served as a loading control.

*Hantavirus L protein exhibits shutoff activity dependent on its N-terminal endonuclease domain*. Given that the N-terminal fragment L_N_250, exhibited robust shutoff activity (Fig. 3C and E), we examined whether this shutoff activity is maintained within the full-length L protein. Unlike L_N_250, which can be expressed from plasmids using either CMV or T7 promoters (Fig. 2B and D), the full-length L protein was only detectable via a T7-driven system (Figs. 1D and 2B). Therefore, the HTNV L protein was expressed from a pT7-IRES plasmid along with firefly luciferase. We found that the expression of the full-length L protein reduced luciferase luminescence by approximately 0.4 log_10_ (Fig. 4A), whereas L_N_250 induced a much more potent suppression of over 1.5 log_10_ (Fig. 3C). Moreover, the L K124A mutant showed lower shutoff activity than WT L (Fig. 4A), indicating that the N-terminal endonuclease-dependent shutoff mechanism is preserved in the context of the full-length L protein, consistent with our observations for L_N_250. These results are consistent with our earlier observation that the expression of WT L, but not the K124A mutant, reduced co-expressed GFP levels (Fig. 1A). Quantitatively, the shutoff activity of the L K124A mutant was 27.1% of the WT L level (Fig. 4B). Similarly, the WT and K124A L proteins from SEOV exhibited the same trend (Fig. 4C), suggesting that the N-terminal endonuclease-dependent shutoff activity of the L protein is conserved among hantaviruses.

Having identified a set of specific mutations, T95A, T112A, and K124A, that modulate shutoff activity at distinct levels in HTNV and SEOV L_N_250 (Fig. 3C, E, F), we determined the impact of these mutations on the shutoff activity and expression of the full-length SEOV L protein. Each mutation reduced the shutoff activity of the L protein by approximately 50%, although no significant differences in shutoff activity were observed among these mutations (Fig. 4D). However, the expression levels of mutant L proteins were inversely correlated with the respective shutoff potencies observed for the corresponding L_N_250 fragment; the K124A, T95A, and T112A mutants exhibited high, moderate, and low protein expression, respectively (Fig. 4E). These data demonstrate that the amino acid residues required for the shutoff activity of L_N_250 are also critical for the function of the full-length L protein to a comparable extent. Furthermore, our findings indicate that the L_N_250 serves as a valuable minimal tool for evaluating the impact of amino acid residues on full-length L protein shutoff activity and expression, as L_N_250 is more amenable to mutagenesis and can be readily expressed using standard Pol II-dependent promoters.

## Discussion

In this study, we demonstrated that hantavirus L proteins possess shutoff activity, which suppress gene expression including their own. Because the N-terminal endonuclease domain involved in viral transcription and replication via the cap-snatching mechanism, is required for the shutoff activity of the L protein, substitution of the key amino acid residues in this domain reduced its shutoff activity as well as enhanced its expression.

The difficulty of expressing hantavirus L proteins has been documented for both Old World and New World hantaviruses^23,24^. Heinemann et al. focused their study on the ANDV L protein using a T7-based expression system, demonstrating that its N-terminus suppresses gene expression and that successful detection requires specific mutations within the endonuclease domain^23^. Similarly, Rothenberger et al. utilized a T7-driven plasmid to show that the N-terminal domains of HTNV and PUUV L proteins mediate cis- and trans-gene suppression^24^. While these studies established the importance of the endonuclease domain within a T7 context, our study extends these findings by performing a systematic comparison of multiple promoter systems. This comprehensive approach revealed that full-length HTNV and SEOV L protein expression is uniquely detectable via the T7 system, a distinction among expression platforms that had not been experimentally addressed in previous reports. Furthermore, our detailed truncation analysis identified specific residues critical for shutoff activity that extend beyond the well-characterized PD-D/ExK motif. The conservation of this mechanism across both HTNV and SEOV, along with our observations in OROV, provides a broad functional context for L protein-mediated shutoff across different bunyavirus families. These identified key residues may serve as critical targets for modulating the expression and functional activity of L proteins in other bunyaviruses, potentially facilitating the development of robust expression systems and reverse genetics across a wider range of related viruses.

By evaluating various expression plasmids with different properties, we found that the shutoff-deficient HTNV L K124A mutant was expressed from the pT7-IRES, pT7-IRES-polyA and pcDNA3.1 plasmids exclusively in the presence of T7 RNA polymerase (Fig. 1B and C). These findings suggest that a T7-driven system, rather than the standard Pol II-dependent expression system, is suitable for the expression of the full-length L protein. In the T7-driven system, T7 RNA polymerase recognizes the T7 promoter to initiate transcription, followed by protein translation in an IRES-dependent, cap-independent manner^25,26^. Although Pol II transcribes RNA with high fidelity through coordination with various eukaryotic factors and is widely used for gene expression in mammalian cells, specific sequences have been reported to induce Pol II pausing or premature termination^27–29^. Furthermore, differences in sequence preference between cap-dependent and IRES-dependent translation have also been documented^30,31^. For instance, high GC content or complex secondary structures that hinder ribosomal scanning, as well as AU-rich sequences frequently found in RNA virus genomes, can induce mRNA degradation or translational stalling in the cap-dependent pathway^32^. In contrast, the IRES-dependent mechanism bypasses the requirement for 5’ cap recognition and ribosomal scanning, potentially allowing for more efficient translation of structurally challenging viral sequences^30,33^. These fundamental mechanistic difference in protein expression likely contribute to the difficulty in expressing the full-length L protein using conventional mammalian expression systems. Further analysis is needed to elucidate the mechanism by which the expression of full-length L protein, particularly within the identified region, remained restricted when using Pol II dependent expression systems.

While influenza A virus segments its RdRp function into three separate subunits, PB2, PB1 and PA, the conserved functional motifs including the endonuclease domain are contained within the single hantavirus L protein^10,11^. By leveraging existing information regarding the amino acid residues involved in the shutoff activity of the influenza PA-X protein^21^, which mediates host shutoff through its N-terminal endonuclease activity, we identified the corresponding amino acid residues of hantavirus L proteins required for their shutoff activity. Specifically, the mutations T95A, T112A, and K124A, which modulate shutoff activity at distinct levels in L_N_250 (Fig. 3C, E and F), also allowed for the modulation of full-length L protein expression levels (Fig. 4E). This set of specific mutations provides a valuable tool for analyzing the interplay between polymerase activity, shutoff activity, and viral replication.

Our data indicate that the hantavirus L protein expressed from plasmids suppresses both *trans-*gene expression and its own (*cis-*) expression (Figs. 1C, 4A, C). However, whether these findings can be recapitulated during viral infection remains unclear. Although the use of recombinant viruses carrying mutant L or the longitudinal monitoring of the L protein levels in infected cells would facilitate the evaluation of the shutoff function of L protein, neither a reverse genetic system nor a highly sensitive antibody capable of detecting the L protein in infected cells is currently available. In influenza A virus, the functions of cap-snatching endonuclease and host shutoff are segregated into the PA protein and its frameshift product, PA-X, respectively^19,20^. Interestingly, our data indicates that L_N_250, comprising the N-terminal 250 amino acids of the L protein, suppresses the luminescence of co-expressed luciferase more strongly than the full-length L protein (Figs. 3C, E, 4A, C), suggesting that this N-terminal fragment exhibits higher shutoff activity than the full-length L protein. Given the absence of a frameshifting motif as described for PA-X or a conserved + 1 ORF corresponding to the X ORF^23^, the hantavirus L protein is currently thought to be responsible for both functions. Nevertheless, the possibility remains that a byproduct comprising the N-terminal region of the L protein could be expressed and involved in host shutoff, independently of the cap-snatching function of the full-length L protein.

The minigenome assay is a powerful tool for evaluating viral polymerase activity. In this analysis, a reporter plasmid is utilized to express either viral RNA (vRNA) or complementary RNA (cRNA) that encodes a reporter gene in place of viral open reading frames (ORFs). These reporter constructs retain the authentic untranslated regions (UTRs), which are essential for recognition by the viral RdRp. Conventionally, the reporter plasmid is co-transfected into cells along with plasmids expressing the L and N proteins, as well as an additional reporter plasmid to normalize the transfection efficiency. The viral polymerase activity is then evaluated by measuring the expression levels of these two reporter genes^22,34^. However, our data indicate that the full-length hantavirus L protein induces endonuclease-mediated shutoff, which suppresses the expression of other genes, including its own (Figs. 1C, 4A, B, 4E). This suggests that during minigenome analysis, the L protein could suppress the expression of the N protein and the normalization control, even if the vRNA or cRNA somehow escapes L-mediated shutoff. Indeed, we confirmed that reporter gene expression from both pCAGGS and pT7-IRES vectors was reduced by the L protein (Figs. 1C, 4A, C). Consequently, the robust shutoff activity of the L protein may lead to an inaccurate assessment of its true polymerase activity when using conventional minigenome systems. Therefore, we emphasize the critical importance of accounting for these *cis-* and *trans-*regulatory effects in functional analyses, such as in minigenome assays, to avoid the misinterpretation of data.

While our study provides a comprehensive analysis of the hantavirus L protein’s shutoff activity and identifies critical residues for its modulation, several limitations should be noted. Our findings are primarily based on plasmid-based expression systems, particularly the T7-driven platform. Although this approach was essential to circumvent the inherent difficulties in expressing the full-length L protein in conventional mammalian systems, the physiological relevance of these findings during actual viral infection remains to be fully elucidated. Currently, the lack of a robust reverse genetics system and highly sensitive antibodies for L protein detection hinders the direct evaluation of this shutoff activity in infected cells. Furthermore, although we characterized the mechanistic differences between Pol II- and T7-dependent systems, the precise cellular factors or sequences that restrict L protein expression in Pol II systems require further investigation.

In summary, we demonstrated that the expression of hantavirus L protein from plasmids in mammalian cells is specifically achieved through a T7-driven system. Given that the L protein possesses shutoff activity that inhibits its own expression, our mutagenesis approach enabled the expression of varying amounts of L proteins with distinct shutoff potencies. Despite the high mortality rate of hantavirus infections, vaccine development remains unestablished^35^, largely due to the lack of a robust reverse genetics system. Our findings presented here will facilitate the expression of both WT and mutant L proteins, providing a crucial foundation for the future development of such systems.

## Methods

*Cells*. BHK-21 (CCL-10, ATCC), BHK-T7^36^, and Human embryonic kidney 293 T (HEK293T) (CRL-3216, ATCC) cells were maintained in DMEM (Wako) containing 10% fetal bovine serum (FBS) (Corning) and penicillin–streptomycin (Gibco). For BHK-T7 cells, 600 µg/ml of hygromycin was added to the medium to maintain the stable expression of T7 RNA polymerase.

*Plasmids*. To construct plasmids for the expression of L proteins of Hantaan virus (HTNV strain 76–118), Seoul virus (SEOV strain SR-11), and Oropouche virus (OROV strain TRVL9760), viral RNA was extracted and reverse-transcribed into cDNA using Superscript IV (Thermo Fisher Scientific) with the following gene-specific primers: TAGTAGTAGACTCCCTAAATAACAAACTCTG (HTNV), TAGTAGTAGACTCCGGAAGAGACAAATC (SEOV), and AGTAGTGTACTCCTATTTCGAAACAAACAAAAAC (OROV). The open reading frames (ORFs) encoding the L proteins were amplified by PCR and cloned into expression vectors with a C-terminal FLAG tag (C-FLAG). Plasmids encoding mutant L proteins or their N-terminal fragments were amplified with appropriate primers and cloned into the same plasmids. All constructs were verified by Sanger sequencing to confirm the nucleotide sequences were identical to the reference sequences. (GenBank accession numbers: KT885047.1 (HTNV), OK500096.1 (SEOV), and MF620129.1 (OROV).)

*Shutoff assay*. 293 T or BHK-T7 cells were seeded in 24-well plates and were co-transfected with a pCAGGS plasmid encoding firefly luciferase together with either an empty vector or a plasmid encoding full-length L protein or its N-terminal fragment (L_N_250) of WT or mutant L protein of HTNV or SEOV. At 48 h post-transfection, luciferase activity was measured using the ONE-Glo Luciferase Assay System (Promega) and a luminometer. The shutoff activity of each L or L_N_250 was calculated by dividing the luminescence value of cells co-transfected with the empty vector by that of cells co-expressing WT L or WT L_N_250.

*Western blotting*. Cells were lysed in 2 × SDS sample buffer at 48 h post-transfection. Whole cell lysates were sonicated, incubated for 10 min at 95 °C, and then loaded onto an 5–20% e-PAGEL (ATTO). Separated proteins were transferred onto PVDF membrane (ThermoFisher) and detected by using anti-DYKDDDDK (FLAG) tag antibody clone 1E6 (FujiFilm), anti-β-actin antibody clone AC-74 (Sigma), or anti-GAPDH antibody clone 10494-1-AP (Proteintech), followed by Goat anti-mouse IgG-HRP (LGC clinical diagnosis) or Goat anti-rabbit IgG-HRP (LGC clinical diagnosis). Full-length images of all blots are provided in Supplementary Fig. S1.

### Statistical analysis

One-way analysis of variance (ANOVA) followed by Dunnett’s test or Tukey’s test and the two-tailed unpaired Student’s *t*-test were performed using GraphPad Prism software. *P* < 0.01 were considered significantly difference. No samples were excluded from the analysis.

## Supplementary Information

Below is the link to the electronic supplementary material.

Supplementary Material 1

## Data Availability

The nucleotide sequences of the L protein ORFs are available in GenBank under the following accession numbers: KT885047.1 (HTNV), OK500096.1 (SEOV), and MF620129.1 (OROV).
