# Supplementary material for: Hantavirus L protein exhibits shutoff activity mediated by its N-terminal endonuclease domain
Source: Sci Rep. 2026 Apr 14;16:17436. doi: 10.1038/s41598-026-47692-3 (PMC13236987; doi:10.1038/s41598-026-47692-3)
Supplement: Supplementary file 1 — Supplementary Material 1 [file 41598_2026_47692_MOESM1_ESM.pdf]

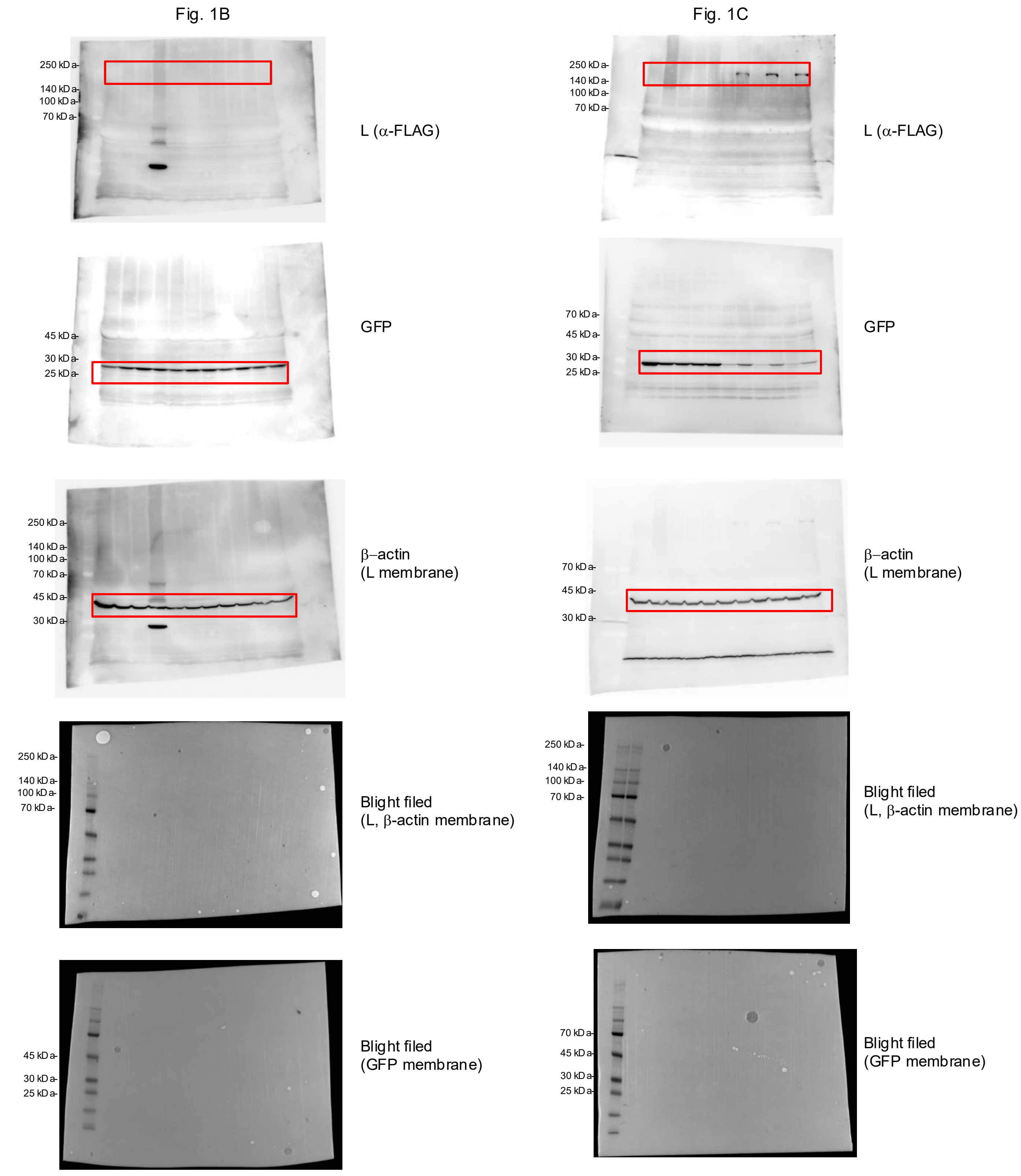

Supplementary Figure S1. Full blot images of all western blots

Fig. 1D

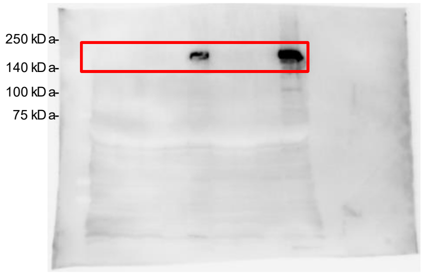

L (α-FLAG)

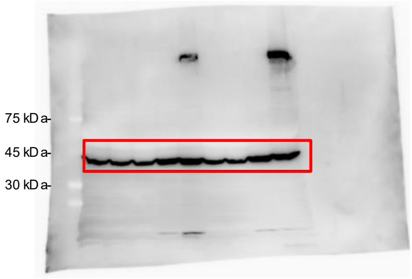

β–actin
(L membrane)

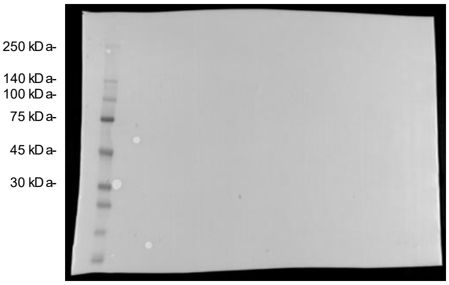

Blight filed

Fig. 1E

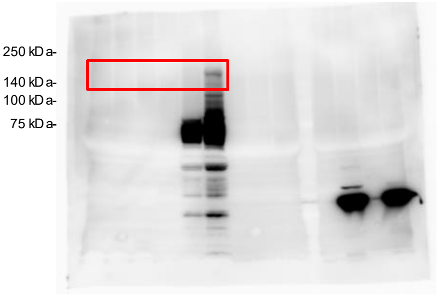

L (α-FLAG)

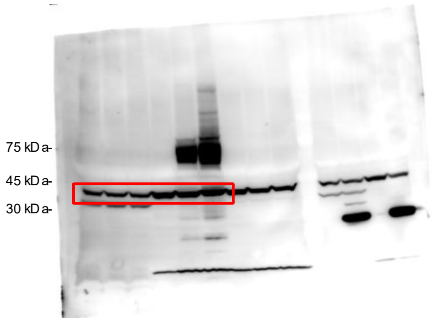

β–actin
(L membrane)

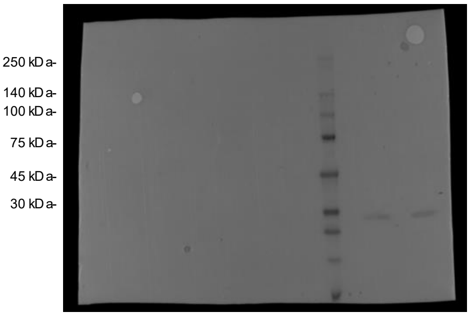

Blight filed

Supplementary Figure S1. Full blot images of all western blots

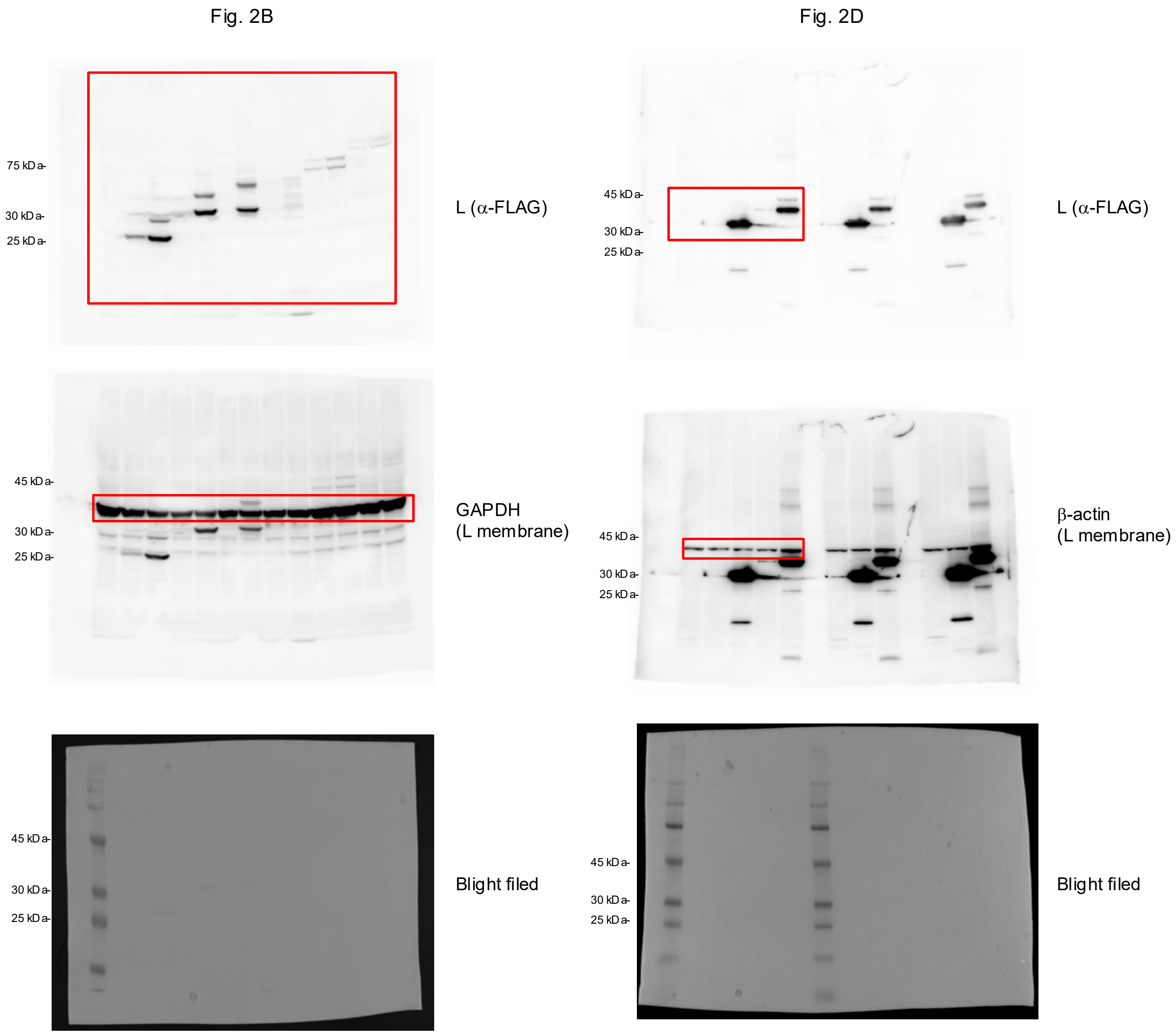

Supplementary Figure S1. Full blot images of all western blots

Fig. 4E

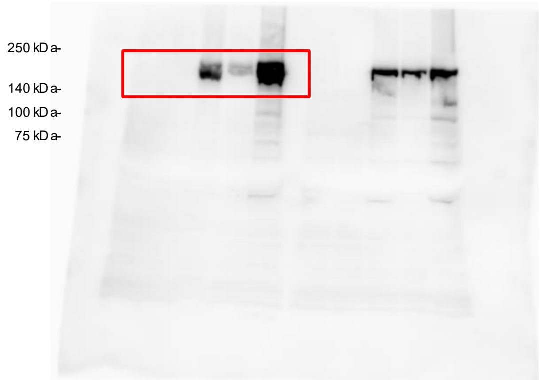

L (α-FLAG)

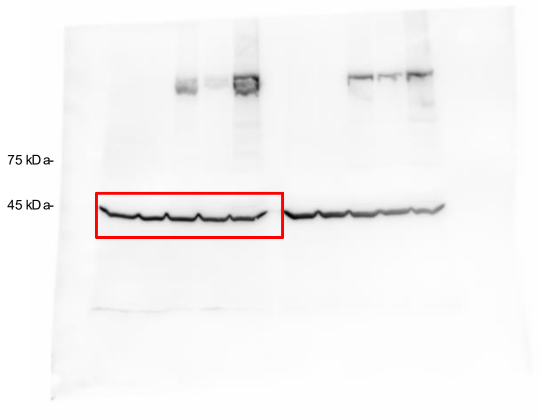

β-actin
(L membrane)

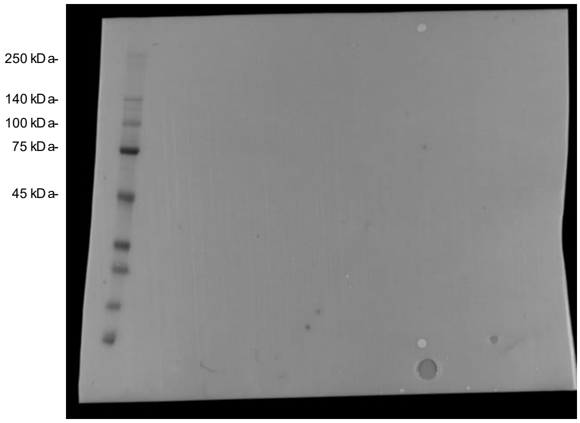

Blight filed

Supplementary Figure S1. Full blot images of all western blots
